# Comprehensive identification and characterization of the HERV-K (HML-9) group in the human genome

**DOI:** 10.1186/s12977-022-00596-2

**Published:** 2022-06-08

**Authors:** Lei Jia, Mengying Liu, Caiqin Yang, Hanping Li, Yongjian Liu, Jingwan Han, Xiuli Zhai, Xiaolin Wang, Tianyi Li, Jingyun Li, Bohan Zhang, Changyuan Yu, Lin Li

**Affiliations:** 1 College of Life Science and Technology, Beijing University of Chemical Technology, Beijing, 100029 China; 2 Department of Virology, Beijing Institute of Microbiology and Epidemiology, Beijing, 100071 China; 3 State Key Laboratory of Pathogen and Biosecurity, Beijing, 100071 China

**Keywords:** Human endogenous retrovirus, HML-9, BLAT, GRCh38/hg38, Gene regulation

## Abstract

**Background:**

Human endogenous retroviruses (HERVs) result from ancestral infections caused by exogenous retroviruses that became incorporated into the germline DNA and evolutionarily fixed in the human genome. HERVs can be transmitted vertically in a Mendelian fashion and be stably maintained in the human genome, of which they are estimated to comprise approximately 8%. HERV-K (HML1-10) transcription has been confirmed to be associated with a variety of diseases, such as breast cancer, lung cancer, prostate cancer, melanoma, rheumatoid arthritis, and amyotrophic lateral sclerosis. However, the poor characterization of HML-9 prevents a detailed understanding of the regulation of the expression of this family in humans and its impact on the host genome. In light of this, a precise and updated HERV-K HML-9 genomic map is urgently needed to better evaluate the role of these elements in human health.

**Results:**

We report a comprehensive analysis of the presence and distribution of HERV-K HML-9 elements within the human genome, with a detailed characterization of the structural and phylogenetic properties of the group. A total of 23 proviruses and 47 solo LTR elements were characterized, with a detailed description of the provirus structure, integration time, potential regulated genes, transcription factor binding sites (TFBS), and primer binding site (PBS) features. The integration time results showed that the HML-9 elements found in the human genome integrated into the primate lineage between 17.5 and 48.5 million years ago (mya).

**Conclusion:**

The results provide a clear characterization of HML-9 and a comprehensive background for subsequent functional studies.

**Supplementary Information:**

The online version contains supplementary material available at 10.1186/s12977-022-00596-2.

## Background

Approximately 45% of the human genome is composed of transposable elements (TEs) [1–3]. Of these, a fraction of TEs are retroelements (REs), which move via a ‘copy and paste’ mechanism involving the reverse transcription of an RNA intermediate and insertion of its cDNA copy at a new position within the host genome [3, 4]. One class of REs, human endogenous retroviruses (HERVs), result from ancestral infections by exogenous retroviruses that became incorporated into the germline DNA and evolutionarily fixed in the genome; HERVs are estimated to comprise approximately 8% of the human genome [5]. HERVs can be transmitted vertically as proviruses in a Mendelian fashion but are not inherently infectious [6–8]. HERVs are structurally similar to the proviruses of common retroviruses, in which the *gag*, *pol*, and *env* genes are flanked by two long terminal repeats (LTRs) that act as promoters [5]. Most HERV families have the *pro* genes, but some families, such as HERV-K HML10, have none [9]. These elements are usually inactive due to the accumulation of substitutions, deletions, and insertions [10, 11]. However, integrated LTR elements have been shown to influence gene regulation by providing regulatory elements such as enhancers, promoters, and splice- and polyadenylation sites for various host genes [4].

HERVs have been divided into three classes, namely, Class I (gamma retrovirus-like), Class II (beta retrovirus-like), and Class III (vaguely spuma retrovirus-like) [12]. The classification of HERVs is complex, with several different classification systems in use. In addition to a system based on *pol* sequence identity, a system based on the tRNA molecule used by retroviruses as a primer during replication is also used. The primer binding site (PBS) regions of Class II HERVs are complementary to lysine (K) tRNA molecules; thus, these HERVs have been designated HERV-Ks [13]. HERV-K proviruses appeared approximately 30–35 million years ago (mya) and are divided into subfamilies from HML-1 through HML-10 [14, 15]. HML-2 of HERV-K, the clade of beta retrovirus-like endogenous retroviruses, is recognized as the most biologically active subgroup, and many of its members still have transcriptional activity [16–20].

The distribution of HERV elements is usually enriched outside transcription units in the human genome. In addition, the few HERV elements within transcription units exhibit a strong orientation bias, such that the orientation of the viral genome is usually opposite to that of host gene transcription. Both of these trends in the location of HERV distribution are likely to be the result of purifying selection. In this case, the harmful HERV provirus within a transcription unit is subject to negative selection and disappears over the course of evolution [12, 15, 21–24]. Because the splicing and poly(A) addition signals of HERV are present in the antisense direction, HERV transcription in the opposite direction to that of the gene may be the least disruptive to mRNA synthesis [15, 21, 22, 25]. A recent study proposed a correlation between silencing mechanisms and the evolutionary age of HERVs. CpG-rich young LTRs were found to be repressed by DNA methylation, while middle-aged LTRs were silenced mainly by posttranslational histone modifications such as H3K9me3 [26].

The first study of the relationship between the expression of the reverse transcriptase (RT) protein of HERV-K and cancer was reported in the early 1970s [27, 28]. Correlations between HERVs and human cancers such as melanoma, breast cancer, germ cell tumors, and ovarian cancer have been described, with significant differences in the protein expression of HERV-K (HML-2/HML-6) in cancer tissues compared to normal tissues. In addition, HERV-K (HML-2) is associated with autoimmune diseases and motor neuron diseases, such as rheumatoid arthritis and amyotrophic lateral sclerosis [29–36]. HERV-K (HML-2) proviruses are classified as type 1 or type 2 based on the presence or absence of a 292 nt deletion at the *pol-env* junction [37]. Type 2 proviruses without these deletions encode Rec or Env. The type 1 provirus with the 292 nt deletion encodes the Np9 protein [38, 39]. Env protein can act as a tumor-specific antigen that impacts both innate and adaptive immune responses, leading to B- and T-cell stimulation and activation and inducing antibody production and cytotoxic T-cell responses [40]. Elevated levels of the Rec or Np9 protein have been observed in breast cancer, ovarian cancer, and leukemia [18, 41, 42]. HERV-H is transcriptionally repressed in adult tissues through DNMT1-dependent cytosine methylation, which contributes to blocks its transcription and translation, potentially triggering an autoimmune response [43, 44]. However, histone deacetylation alone is not responsible for the repression of HERV family members (HERV-K (HML-2), HERV-W, HERV-FRD), and HDACi treatment did not significantly upregulate HERVs in either latent cell lines or primary T cells infected with HIV-1 [45].

Characterization of the genomic distribution of the HML-9 group is critical to understanding the regulation of the expression of this family and its relationship with human health and disease. To date, there has been only limited characterization of and research on HML-9. In light of this, a precise and updated HML-9 genomic map is urgently needed to better evaluate the role of these elements in human health.

Here, we report a comprehensive analysis of the presence and distribution of HERV-K HML-9 elements within the human genome, with a detailed characterization of the structural and phylogenetic properties of the group. Additionally, we analyzed the provirus integration time and the genes that may be  regulated by these elements. Overall, the results provide a clear characterization of HML-9 and a comprehensive background for subsequent functional studies.

## Materials and methods

### HML-9 identification, localization, and genomic distribution

To evaluate the HML-9 provirus and solo LTR distribution in the human genome, we performed HML-9 identification by using the Genome Reference Consortium assembly GRCh38/hg38 (released Dec. 2013) as the human genome reference. A traditional BLAT search tool [46] in the UCSC Genome Browser database [47] was used to identify the integrated HML-9 elements. DNA BLAT works by keeping an index of the entire genome in its memory. The index consists of all overlapping 11-mers stepped by 5, except for those heavily involved in repeats (http://genome.ucsc.edu/cgi-bin/hgBlat). The assembled LTR14C-HERVK14C-LTR14C sequence was used as a query. Generally, there are two resources that can be selected as references: consensus representatives or single best representative strains. The major advantage of consensus representatives is their much broader representation [9, 48]. Therefore, they are used as references or queries in most studies. The assembled LTR14C-HERVK14C-LTR14C in the current work is from the Dfam database. Additionally, the expected distribution of the HML-9 loci on each chromosome was predicted according to the formula e = Cl × n/Tl (e is the number of integrations expected in the chromosome, Cl represents the ungapped length of the chromosome, n is the total number of actual HML-9 loci identified in the human genome, and Tl represents the sum ungapped length of all chromosomes) [49]. The difference between the expected number of integrations and the actual number of HML-9 loci was analyzed via the chi-square (χ^2^) test, and statistical significance was estimated according to the *p* value.

### Structural characterization

The identified HML-9 elements were aligned to the proviral reference LTR14C-HERVK14C-LTR14C. Alignments were analyzed on the BioEdit software platform [50]. All insertions and deletions were annotated.

### Phylogenetic analyses

Maximum likelihood (ML) phylogenetic trees were built with MEGA7 [51] to confirm the assignment of the identified HML-9 elements. The 44 out of 47 solo LTR sequences that were longer than 90% of LTR14C and the 5 out of 23 proviral sequences that were longer than 80% of LTR14C-HERVK14C-LTR14C were used to construct phylogenetic trees. The best-fit models of nucleotide substitution for solo LTRs and full-length proviruses were calculated as K2 + G and GTR + G + I by the Model Selection function in MEGA7, respectively. For the 4 coding regions, elements longer than 90% of the corresponding section of HML-9 were screened to construct phylogenetic trees. According to the model selection function of MEGA7, the best-fit models of nucleotide substitution for *gag*, *pro*, *pol*, and *env* analysis were HKY + G + I, GTR + G + I, GTR + G, and HKY + G, respectively. Tree topologies were searched using the nearest neighbor interchange (NNI) procedure. The confidence of each node in phylogenetic trees was determined using bootstrap testing with 500 bootstrap replicates. The final ML trees were visualized using iTOL [52].

### Estimation of the integration time of HML-9

To estimate the time of integration, we used the substitution rate of 0.2%/nucleotide/million years to assess the effect of divergence on each HML-9 element [53]. D is the percentage of divergent nucleotides, and the D of each HML-9 member was estimated between (1) the 5′ and 3′ LTRs of each provirus and (2) each HML-9 internal element (*gag*, *pro*, *pol*, and *env* genes) and its generated consensus. The divergence values were estimated with MEGA7. For the 4 internal regions, the integration time was calculated based on the formula T = D/0.2, in which T represents the estimated time of integration (in million years). For the flanking LTR regions, the provirus integration time was calculated based on the formula T = D/0.2/2.

### Functional prediction of cis-regulatory regions and enrichment analysis

Noncoding regions typically lack biological function annotations. To understand the biological significance of both HML-9 solo LTRs and proviral LTRs, an analysis of the annotations of genes adjacent to LTRs was performed based on the Genomic Regions Enrichment of Annotations Tool (GREAT) [54]. The association rule was as follows: basal + extension: 5000 bp upstream, 1000 bp downstream; 1,000,000 bp max extension; curated regulatory domains included. After identifying potential regulatory genes, the WEB-based Gene SeT AnaLysis Toolkit (WebGestalt) [55] was used to analyze their functional enrichment (http://www.webgestalt.org), which is crucial for interpreting the list of genes of interest. The enrichment method used in the current work was over-representation analysis (ORA). The parameters for the enrichment analysis were as follows: minimum number of IDs in the category: 5; maximum number of IDs in the category: 2000; FDR Method: Benjamini–Hochberg (BH); and significance level: top 10.

### In silico examination of conserved transcription factor binding sites

The transcription factor binding sites of the HML-9 LTR consensus reference sequence were predicted from the JASPAR (https://jaspar.genereg.net/) database. The taxon was vertebrates, and the species was *Homo sapiens*. The data chosen for the prediction of transcription factors were ChIP-seq data in JASPAR with a relative profile score threshold of 95%. The alignment and annotation of the HML-9 LTR reference sequence with the 4 proviral sequences (length of 5′LTR > 90%) were performed using Geneious software [56].

### Primer binding site feature representation

The composition of the primer binding sites (PBSs) of 11 near-full-length proviruses (LTR length > 80%) and the HML-9 reference sequence were all analyzed using MEGA7 and BioEdit. The degree of conservation at each position was represented by a logo built from WebLogo at http://weblogo.berkeley.edu [57]. Then, the PBS type was identified with tRNAdb (http://trna.bioinf.uni-leipzig.de/) [58].

## Results

### HML-9 element identification, localization, and distribution in hg38

**Fig. 1:**
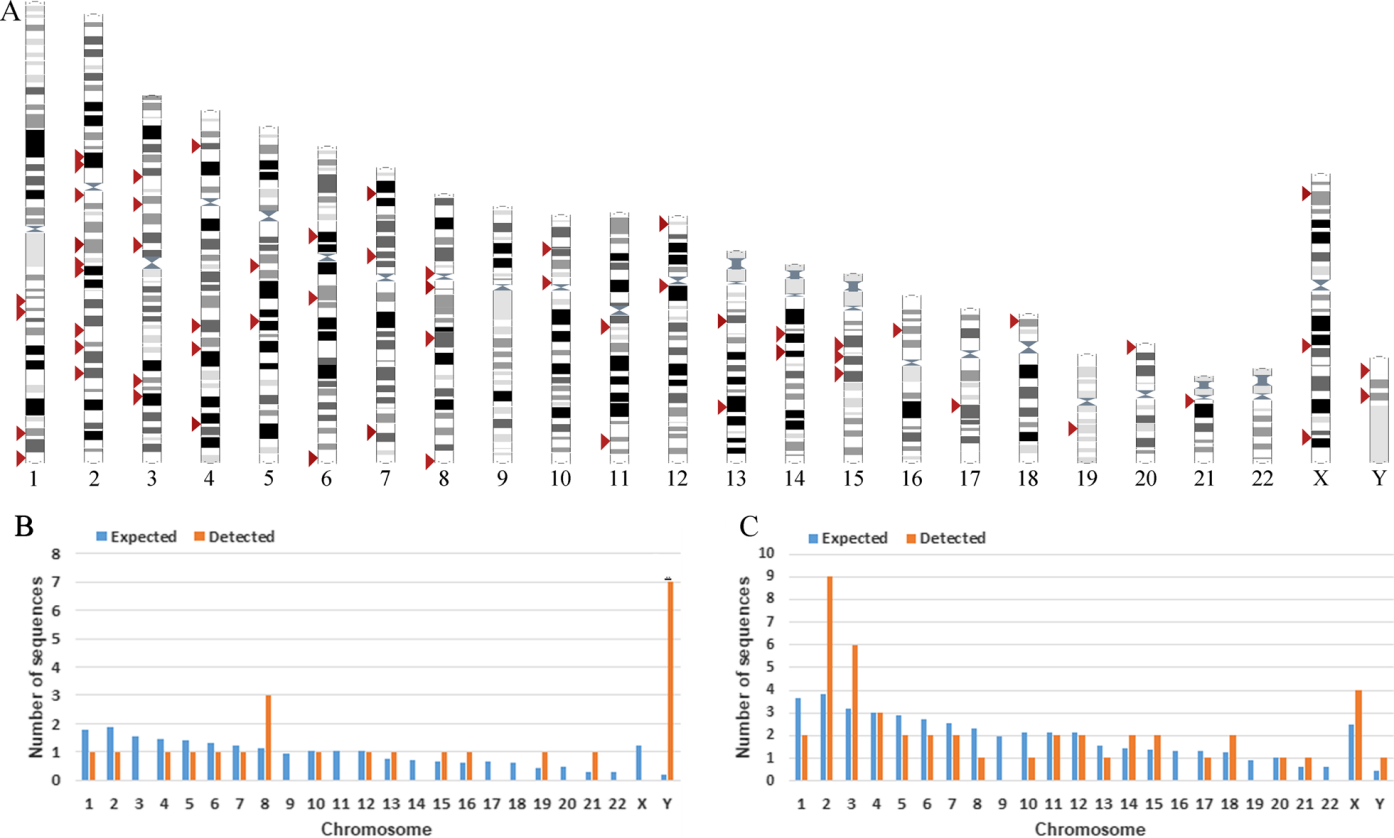
Chromosomal distribution of HML-9 loci. **A** All HML-9 elements (red arrows) are displayed on the human karyotype (www.ensembl.org). The number of HML-9 proviral elements (**B**) and solo LTRs (**C**) integrated into each human chromosome was determined and compared to the expected number of insertion events. The expected number of sequences in each chromosome is marked in blue, and the actual number of sequences detected is marked in orange

According to the BLAT results for LTR14C-HERVK14C-LTR14C in GRCh38/hg38, we characterized a total of 23 HERV-K HML-9 proviral elements. Each HML-9 element was named according to the genomic locus of integration, as a previously proposed nomenclature for HERV-Ks [16] (Table 1). Element length analysis indicated that 6 elements were longer than 70% of the full length of the reference, 9 elements were between 40 and 70% of the reference length, and the remaining 8 elements were between 17.11 and 34.26% of the reference length. Moreover, a total of 47 solo LTR elements of HERV-K HML-9 were characterized. Of these, 44 solo LTRs (93.62%) were longer than 90% of the representative reference LTR14C. The nucleotide sequence of each element is shown in Additional file 1: Dataset S1, Additional file 2: Dataset S2. The overall HML-9 element distribution is displayed based on the data representation obtained from the Ensemble website (www.ensembl.org) (Fig. 1A).

**Table 1:** HML-9 provirus distribution

Number	Locus	Chromosome	Strand	Position start	Position end	Length (bp)	Match + mismatch (bp)/full length (bp) (%)	Range (%)	Qgap (bp)/match + mismatch + Qgap (bp) (%)	Insertion or deletion	Intergenic/intron/exon	Gene including the region
1	16p12.3	chr16	−	19393581	19402152	8572	96.00	(90–100)	1.01	NA	Exon_intron	AC130456.2
2	2p12	chr2	+	82022660	82031279	8620	95.91	(90–100)	1.13	NA	Intergenic	NA
3	15q21.1	chr15	−	45234477	45243073	8597	95.34	(90–100)	1.85	NA	Exon_intron	AC051619.4
4	8p11.1	chr8	−	43694016	43702583	8568	95.10	(90–100)	2.14	NA	Intergenic	NA
5	13q31.1	chr13	+	84869526	84877320	7795	86.84	(80–90)	6.67	NA	Exon_intron	AL445588.1
6	4q33	chr4	−	170126345	170133883	7539	70.03	(70–80)	0.79	Insertion	Intergenic	NA
7	6p12.3	chr6	+	48873675	48879725	6051	64.84	(60–70)	34.48	Deletion	Intergenic	NA
8	Yp11.2	chrY	−	9273707	9279611	5905	59.83	(50–60)	39.23	Deletion	Intergenic	NA
9	8q24.3	chr8	+	145019974	145032719	12,746	57.06	(50–60)	0.79	Insertion	Intergenic	NA
10	Yq11.223	chrY	+	21580120	21585551	5432	57.04	(50–60)	38.30	Deletion	Exon_intron	TTTY13
11	19q13.2	chr19	+	40954172	40959178	5007	56.66	(50–60)	41.93	Deletion	Intergenic	NA
12	Yp11.2	chrY	−	8121821	8126768	4948	54.57	(50–60)	44.92	Deletion	Intergenic	NA
13	Yp11.2	chrY	+	8996062	9000755	4694	50.80	(50–60)	41.95	Deletion	Intergenic	NA
14	Yq11.222	chrY	−	18622534	18626952	4419	47.33	(40–50)	52.23	Deletion	Intergenic	NA
15	Yq11.223	chrY	−	21845475	21850069	4595	43.18	(40–50)	49.78	Deletion/insertion	Exonic_intergenic	AC024236.1
16	21q21.1	chr21	−	18563368	18566735	3368	34.26	(30–40)	8.19	NA	Exon_intron	MIR548XHG
17	5q33.3	chr5	−	156660448	156663815	3368	34.14	(30–40)	8.50	NA	Intron	SGCD
18	1q22	chr1	−	155629408	155632775	3368	33.75	(30–40)	9.56	NA	Intron	AL353807.5
19	7q36.1	chr7	−	150561277	150563994	2718	27.92	(20–30)	10.27	Deletion	Intergenic	NA
20	8q21.13	chr8	+	78652302	78654820	2519	26.60	(20–30)	0.30	NA	Intron	AC068700.2
21	10q24.2	chr10	−	99822511	99825532	3022	25.36	(20–30)	24.65	Deletion/insertion	Intron	ABCC2
22	12q13.11	chr12	+	48509228	48511681	2454	18.44	(10–20)	33.18	Deletion/insertion	Intergenic	NA
23	Yq11.222	chrY	−	17669948	17671523	1576	17.11	(10–20)	12.69	Deletion	Intergenic	NA

Next, the expected number of integrations of HML-9 elements per chromosome was predicted and compared with the number of actually detected sites to assess whether HML-9 is randomly distributed in the human genome. The number of HML-9 integration events observed was often inconsistent with the expected number (Fig. 1B, C). For the proviral elements, the number of HML-9 insertions on chromosomes 8, 13, 15, 16, 19, 21, and Y was higher than expected. In particular, the number of proviral elements on the Y chromosome was significantly higher than that predicted by the chi-square test (p < 0.05). On chromosomes 1, 2, 4, 5, 6, 7, 10, and 12, the actual numbers identified were lower than expected (Fig. 1B). Notably, no HML-9 provirus integration was detected on chromosomes 3, 9, 11, 14, 17, 18, 20, 22, and X. With respect to the solo LTR elements, the number of HML-9 solo LTRs on chromosomes 2, 3, 14, 15, 18, 21, X, and Y was higher than expected. On chromosomes 1, 4, 5, 6, 7, 8, 10, 11, 12, 13, 17, and 20, the actual numbers identified were lower than expected. In particular, no HML-9 solo LTR integration was detected on chromosomes 9, 16, 19, and 22 (Fig. 1C). This analysis revealed that HML-9 provirus and solo LTR integration is nonrandom among human chromosomes.

Furthermore, all 23 identified proviral elements and 47 solo LTRs were analyzed to determine their locations in intergenic regions, introns, or exons (Tables 1, 2). The results showed that 13 proviral elements were located in intergenic regions, accounting for 56.52% of all proviral elements. Four proviral elements (17.39%) were located in introns. Six proviral elements (26.09%) were located in both introns and exons (Table 1). With respect to solo LTRs, 28 (59.57%) were located in intergenic regions, and the remaining 19 (40.43%) were located in introns (Table 2). The results revealed an apparent distribution preference for intergenic regions and introns. Previously, Brady et al. [15] demonstrated that the accumulation of HML-2 proviruses in introns and intergenic regions is not a result of integration bias but selection against proviruses that integrate into exons and genic regions. This conclusion also applies to the current study. The proviruses in genes and their relative transcriptional orientation are presented in Additional file 3: Table S1, Additional file 4: Table S2.

**Table 2:** HML-9 solo LTR tracks distribution

Number	Locus	Chromosome	Strand	Position start	Position end	Length (bp)	Percentage of LTR14C in length (%)	Match + mismatch/full length (%)	Range (%)	Qgap (bp)/match + mismatch + Qgap (bp) (%)	Insertion or deletion	Intergenic/intron/exon	Gene including the region
1	14q21.1	chr14	+	38011040	38012012	973	101.36	6.91	(0–10)	35.61	Deletion, insertion	Intron	TTC6
2	Xq21.32	chrX	−	93273183	93274197	1015	100.85	6.88	(0–10)	2.47	Insertion	Intergenic	NA
3	2q31.1	chr2	+	180236847	180237437	591	100.34	6.84	(0–10)	0.34	NA	Intergenic	NA
4	18p11.31	chr18	–	4527618	4528209	592	100.17	6.83	(0–10)	0.00	NA	Intergenic	NA
5	2q11.2	chr2	+	97964920	97965508	589	100.00	6.82	(0–10)	0.00	NA	Intron	TMEM131
6	15q14	chr15	+	39011033	39011621	589	100.00	6.82	(0–10)	0.00	NA	Intron	LINC02694
7	2p12	chr2	–	81304430	81305068	639	99.83	6.81	(0–10)	0.00	NA	Intergenic	NA
8	2q32.3	chr2	–	194256159	194256746	588	99.83	6.81	(0–10)	0.00	NA	Intergenic	NA
9	3q26.1	chr3	–	163283189	163283777	589	99.83	6.81	(0–10)	0.00	NA	Intron	LINC01192
10	4q26	chr4	+	116980222	116980809	588	99.83	6.81	(0–10)	0.34	NA	Intergenic	NA
11	4p15.31	chr4	+	19556097	19556684	588	99.83	6.81	(0–10)	0.17	NA	Intron	AC024230.1
12	7p21.2	chr7	+	14509240	14509827	588	99.83	6.81	(0–10)	0.00	NA	Intron	DGKB
13	8q11.21	chr8	–	51178592	51179179	588	99.83	6.81	(0–10)	0.00	NA	Intergenic	NA
14	11q12.3	chr11	–	62185237	62185824	588	99.83	6.81	(0–10)	0.00	NA	Intergenic	NA
15	2q21.3	chr2	+	135521883	135522470	588	99.66	6.80	(0–10)	0.17	NA	Intron	ZRANB3
16	3p12.2	chr3	–	81329902	81330488	587	99.66	6.80	(0–10)	0.17	NA	Intergenic	NA
17	3p12.1	chr3	–	83618409	83618995	587	99.66	6.80	(0–10)	0.17	NA	Intergenic	NA
18	5q21.3	chr5	–	105998962	105999549	588	99.66	6.80	(0–10)	0.34	NA	Intergenic	NA
19	10p12.31	chr10	–	18856645	18857233	589	99.66	6.80	(0–10)	0.17	NA	Intergenic	NA
20	Yq11.23	chrY	–	25974734	25975320	587	99.66	6.80	(0–10)	0.17	NA	Intergenic	NA
21	6q14.1	chr6	–	82297755	82298498	744	99.49	6.78	(0–10)	0.34	Insertion	Intergenic	NA
22	Xp22.2	chrX	+	11033746	11034330	585	99.32	6.77	(0–10)	0.51	NA	Intron	AC073529.1
23	2p12	chr2	+	77807602	77808185	584	99.15	6.76	(0–10)	0.68	NA	Intron	AC012494.1
24	1q23.3	chr1	+	162419359	162419942	584	98.98	6.75	(0–10)	0.17	NA	Intergenic	NA
25	Xq27.2	chrX	–	142767872	142768454	583	98.98	6.75	(0–10)	0.00	NA	Intergenic	NA
26	2q31.1	chr2	+	171365032	171365617	586	98.64	6.73	(0–10)	1.19	NA	Intron	METTL8
27	5q13.3	chr5	+	75859521	75860102	582	98.64	6.73	(0–10)	0.17	NA	Intergenic	NA
28	Xq27.3	chrX	–	144791258	144791846	589	98.47	6.71	(0–10)	1.37	NA	Intergenic	NA
29	12q12	chr12	+	38144469	38145052	584	98.30	6.70	(0–10)	1.03	NA	Intergenic	NA
30	15q21.3	chr15	–	54594796	54595373	578	98.13	6.69	(0–10)	2.04	NA	Intron	UNC13C
31	21q11.2	chr21	–	14080466	14081052	587	97.79	6.67	(0–10)	2.05	NA	Intron	AP001347.1
32	4q28.2	chr4	+	129080872	129081454	583	97.61	6.66	(0–10)	2.22	NA	Intron	SCLT1
33	3q25.2	chr3	–	154944330	154944911	582	97.44	6.64	(0–10)	2.56	NA	Intergenic	NA
34	11q24.2	chr11	+	124270705	124271275	571	96.93	6.61	(0–10)	0.00	NA	Intergenic	NA
35	2q14.3	chr2	–	125024208	125024792	585	96.76	6.60	(0–10)	3.07	NA	Intergenic	NA
36	6q27	chr6	+	169084226	169084808	583	96.76	6.60	(0–10)	3.07	NA	Intergenic	NA
37	13q13.3	chr13	–	38319721	38320300	580	96.76	6.60	(0–10)	3.24	NA	Intron	LINC00571
38	7q35	chr7	+	143472173	143472744	572	96.08	6.55	(0–10)	3.75	NA	Intron	EPHA1-AS1
39	14q21.3	chr14	+	48011215	48011780	566	95.91	6.54	(0–10)	0.53	NA	Intergenic	NA
40	3p21.31	chr3	+	44534488	44535059	572	95.74	6.53	(0–10)	3.77	NA	Intergenic	NA
41	2q22.1	chr2	–	138860917	138861512	596	95.06	6.48	(0–10)	3.79	NA	Intergenic	NA
42	12p13.32	chr12	–	4720007	4720593	587	94.89	6.47	(0–10)	4.95	NA	Intron	AC005833.1
43	3p14.2	chr3	–	59469489	59470030	542	91.82	6.26	(0–10)	3.23	NA	Intron	AC126121.3
44	1q24.2	chr1	+	168457190	168457732	543	90.80	6.19	(0–10)	0.37	NA	Intron	AL023755.1
45	20p13	chr20	–	2809052	2809886	835	88.42	6.03	(0–10)	0.38	Insertion	Intergenic	NA
46	18q21.33	chr18	+	63648105	63648555	451	76.49	5.22	(0–10)	0.22	NA	Intron	SERPINB11
47	17q22	chr17	+	52961655	52962071	417	70.87	4.83	(0–10)	0.24	NA	Intergenic	NA

### Structural characterization

**Fig. 2:**
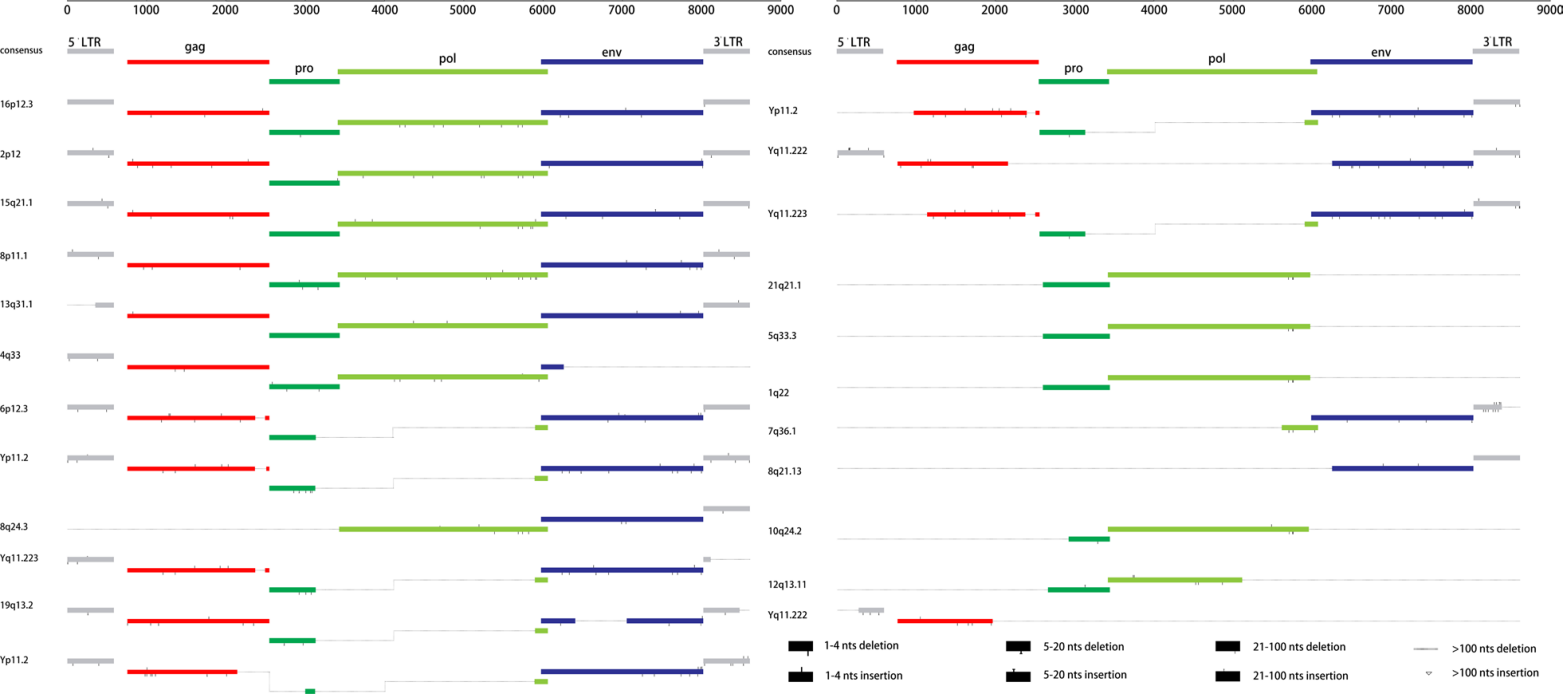
HML-9 provirus structural characterization. Each HML-9 provirus element was analyzed and compared to the Dfam reference sequence. The LTRs and the *gag*, *pro*, *pol*, and *env* genes were annotated. Black lines represent deleted regions

To define the structural characteristics of HML-9 elements, the 23 proviruses were further analyzed by comparing them with the reference LTR14C-HERVK14C-LTR14C. According to the annotation information summarized in the Dfam database (https://www.dfam.org/family/DF0000193/features), the complete HML-9 reference exhibits a typical proviral structure, containing 4 open reading frames (ORFs) and 2 flanking LTRs. Specifically, the 5′ LTR is located from nucleotides 1 to 587, the CDS range of the HERVK14C_gag protein is from nucleotides 758 to 2548, the CDS range of the HERVK14C_pro protein is from nucleotides 2548 to 3435, the CDS range of the HERVK14C_pol protein is from nucleotides 3411 to 6060, the CDS range of the HERVK14C_env protein is from nucleotides 5975 to 8020, and the 3′ LTR is from nucleotides 8022 to 8608. We aligned the 23 HML-9 proviral sequences and annotated the position of the single retroviral component and deletions to describe the structure of each HML-9 provirus element (Fig. 2). HML-9 16p12.3, 2p12, 15q21.1, 8p11.1, 13q31.1 and 4q33 are longer than 70% of the complete reference sequence in length. Furthermore, the integrity of 6 separate regions (5′ LTR, *gag*, *pro*, *pol*, *env*, and 3’ LTR) is summarized in Table 3.

**Table 3:** The integrity of 6 separate regions relative to the corresponding sections of reference

Number	Locus	Provirus regions	5′LTR (%)	gag (%)	pro (%)	pol (%)	env (%)	3′LTR (%)
1	16p12.3	chr16 19393581 19402152	100.00	99.83	99.89	99.39	99.17	99.66
2	2p12	chr2 82022660 82031279	98.98	99.72	99.89	99.43	99.90	99.15
3	15q21.1	chr15 45234477 45243073	99.83	99.27	100.00	99.66	99.56	99.83
4	8p11.1	chr8 43694016 43702583	99.83	99.44	98.31	99.39	99.66	99.83
5	13q31.1	chr13 84869526 84877320	35.78	99.55	52.20	99.77	99.80	99.32
6	4q33	chr4 170126345 170133883	99.66	99.89	99.77	99.70	13.93	0.00
7	6p12.3	chr6 48873675 48879725	99.15	92.79	65.84	6.13	99.85	99.66
8	Yp11.2	chrY 9273707 9279611	88.42	90.73	63.81	6.36	98.83	99.49
9	8q24.3	chr8 145019974 145032719	0.00	0.00	0.79	99.77	99.90	95.23
10	Yq11.223	chrY 21580120 21585551	88.25	91.74	64.83	6.25	99.36	15.84
11	19q13.2	chr19 40954172 40959178	98.98	98.94	64.26	6.17	67.40	77.51
12	Yp11.2	chrY 8121821 8126768	99.66	76.49	14.09	6.40	99.17	98.81
13	Yp11.2	chrY 8996062 9000755	0.00	80.23	64.37	6.47	99.80	95.55
14	Yq11.222	chrY 18622534 18626952	96.76	75.21	0.00	0.00	84.75	98.47
15	Yq11.223	chrY 21845475 21850069	0.00	70.85	64.60	6.40	99.32	99.15
16	21q21.1	chr21 18563368 18566735	0.00	0.00	95.26	96.12	0.00	0.00
17	5q33.3	chr5 156660448 156663815	0.00	0.00	95.26	96.12	0.00	0.00
18	1q22	chr1 155629408 155632775	0.00	0.00	95.26	96.12	0.00	0.00
19	7q36.1	chr7 150561277 150563994	0.00	0.00	0.00	16.91	99.02	54.51
20	8q21.13	chr8 78652302 78654820	0.00	0.00	0.00	0.00	87.34	100.00
21	10q24.2	chr10 99822511 99825532	0.00	0.00	52.29	95.43	0.00	0.00
22	12q13.11	chr12 48509228 48511681	0.00	0.00	88.72	63.52	0.00	0.00
23	Yq11.222	chrY 17669948 17671523	52.81	62.09	0.00	0.00	0.00	0.00

### Phylogenetic analyses

**Fig. 3:**
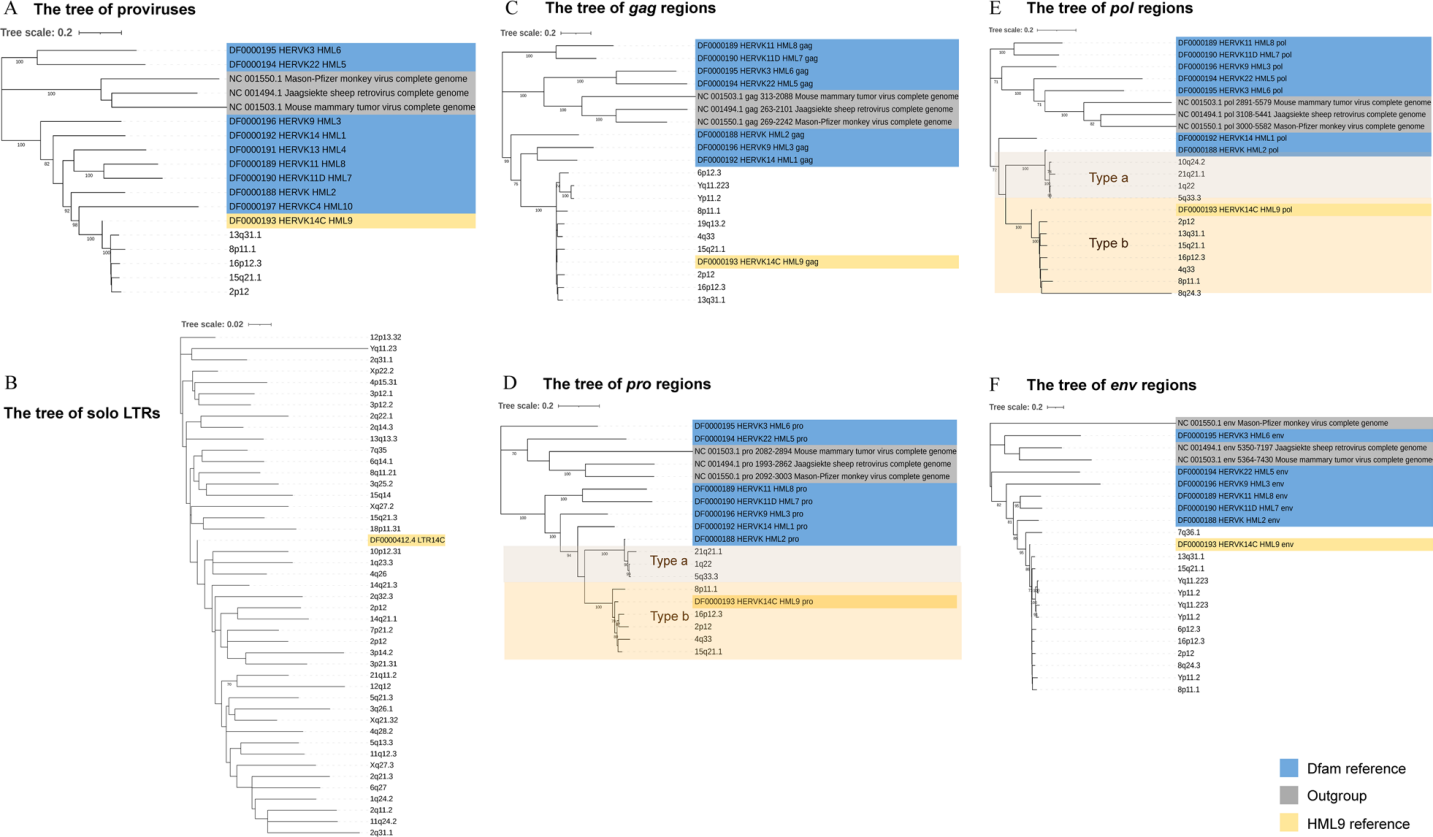
Phylogenetic analysis of the HML-9 near-full-length proviruses, solo LTRs, and 4 subregions by the maximum likelihood method. Phylogenetic analyses of 5 HML-9 proviral elements (**A**), 44 solo LTRs (**B**), 10 *gag* elements (**C**), 8 *pro* elements (**D**), 11 *pol* elements (**E**), and 13 *env* elements (**F**), together with references. The two intragroup clusters of the *pro* and *pol* genes (types a and b) were annotated and depicted with brown and orange background colors, respectively. The resulting phylogeny was tested by the bootstrap method with 500 replicates. The branch length indicates the number of substitutions per site

To characterize the phylogenetic relationships among the HML-9 group, 5 proviral sequences (longer than 80% of the HML-9 reference length) were screened together with Dfam HERV-K groups (HML-1–10) and exogenous betaretroviruses as representatives to generate ML trees. The 5 identified proviruses all clustered with the Dfam HML-9 reference by 100% of bootstrap support (Fig. 3A). Subsequently, phylogenetic trees of a total of 44 solo LTRs identified to be longer than 90% of LTR14C were constructed together with the LTR reference (Fig. 3B). Next, 4 ML trees were constructed for subregions whose lengths were longer than 90% of the corresponding section of the reference sequence, including 10 *gag* elements, 8 *pro* elements, 11 *pol* elements, and 13 *env* elements (Fig. 3C–F). These phylogenetic groups of different regions of HML-9 all clustered together and were clearly separated from the other HERV-K groups (HML1-8, 10). Two distinct clusters in the *pro* and *pol* groups were observed. They were statistically supported by 100% of bootstrap values and were named HML-9 type a and type b. The results showed that HML-9 21q21.1, HML-9 1q22, and HML-9 5q33.3 were included in HML-9 type a, whereas HML-9 15q21.1, HML-9 16p12.3, HML-9 4q33, HML-9 8p11.1, and HML-9 2p12 were included in HML-9 type b. HML-9 type b sequences included the Dfam HML-9 reference, whereas HML-9 type a elements showed more divergence relative to the group references.

### Estimated time of integration

The HML-9 proviral age in the human genome was estimated based on the LTRs and the *gag*, *pro*, *pol*, and *env* regions. Each region whose length exceeded 90% of the corresponding reference sequence was selected to calculate the integration time. For LTRs, the 5′ and 3′ LTRs of a given provirus are identical at the time of integration and then accumulate random substitutions in an independent manner [53]; therefore, the T value was estimated by the relation T = D/0.2/2. For the *gag*, *pro*, *pol*, and *env* regions, the ancestral sequences of each region were generated via MEGA7 following the ML method based on multiple alignments of all elements. The T value (integration time) was estimated by the relation T = D/0.2, where 0.2 represents the human genome neutral mutation rate expressed in substitutions/nucleotide/million years. For each provirus region, we provide details on the period of provirus formation in Table 4. Overall, the estimated time of integration based on LTR elements is later than that estimated based on the four regions (*gag*, *pro*, *pol*, and *env*). The LTRs integrated between 17.5 and 48.5 mya. The average time of integration was 28.83 mya. However, the majority of HML-9 elements (*gag*, *pro*, *pol*, and *env*) found in the human genome integrated between 37.5 and 151.5 mya. The average time of integration was 76 mya. There exists a very large discrepancy between the two analyses. A reasonable explanation for the difference between the two methods is as follows. The two flanking LTRs (5' LTR and 3' LTR) were identical when the provirus was integrated into the host genome. However, the internal regions contain multiple sequence differences due to the mutations accumulated during viral replication cycles, with a much higher error rate. This difference would inevitably lead to LTRs being a more accurate timing starting point for integration time estimation.

**Table 4:** Estimated time of HML-9 elements integration

Locus	Provirus regions	Divergence from consensus sequence				Mean divergences	*T* = *D*/0.2	Age/million years (gene vs consensus)	Divergence between 2 LTRs	T = D/0.2/2	Age/million years (LTR vs LTR)
		gag	pro	pol	env						
16p12.3	chr16 19393581 19402152	0.059	0.158	0.206	0.089	0.128	0.64	64.00	0.082	0.20500	20.50000
2p12	chr2 82022660 82031279	0.061	0.182	0.204	0.101	0.137	0.685	68.50	0.070	0.17500	17.50000
15q21.1	chr15 45234477 45243073	0.051	0.126	0.206	0.099	0.121	0.6025	60.25	0.080	0.20000	20.00000
8p11.1	chr8 43694016 43702583	0.091	0.177	0.231	0.121	0.155	0.775	77.50	0.107	0.26750	26.75000
13q31.1	chr13 84869526 84877320	0.054	NA	0.208	0.103	0.122	0.608333333	60.83	NA	NA	NA
4q33	chr4 170126345 170133883	0.058	0.172	0.214	NA	0.148	0.74	74.00	NA	NA	NA
6p12.3	chr6 48873675 48879725	0.063	NA	NA	0.106	0.085	0.4225	42.25	0.110	0.27500	27.50000
Yp11.2	chrY 9273707 9279611	0.114	NA	NA	0.139	0.127	0.6325	63.25	0.141	0.35250	35.25000
8q24.3	chr8 145019974 145032719	NA	NA	0.513	0.093	0.303	1.515	151.50	NA	NA	NA
Yq11.223	chrY 21580120 21585551	0.105	NA	NA	0.140	0.123	0.6125	61.25	NA	NA	NA
19q13.2	chr19 40954172 40959178	0.075	NA	NA		0.075	0.375	37.50	0.097	0.24250	24.25000
Yp11.2	chrY 8121821 8126768	NA	NA	NA	0.125	0.125	0.625	62.50	0.157	0.39250	39.25000
Yp11.2	chrY 8996062 9000755	NA	NA	NA	0.133	0.133	0.665	66.50	NA	NA	NA
Yq11.222	chrY:18622534–18626952	NA	NA	NA	NA	NA	NA	NA	0.194	0.48500	48.50000
Yq11.223	chrY 21845475 21850069	NA	NA	NA	0.143	0.143	0.715	71.50	NA	NA	NA
21q21.1	chr21 18563368 18566735	NA	0.266	0.190	NA	0.228	1.14	114.00	NA	NA	NA
5q33.3	chr5 156660448 156663815	NA	0.215	0.160	NA	0.188	0.9375	93.75	NA	NA	NA
1q22	chr1 155629408 155632775	NA	0.212	0.156	NA	0.184	0.92	92.00	NA	NA	NA
7q36.1	chr7 150561277 150563994	NA	NA	NA	0.197	0.197	0.985	98.50	NA	NA	NA
10q24.2	chr10 99822511 99825532	NA	NA	0.169	NA	0.169	0.845	84.50	NA	NA	NA

### Functional prediction of cis-regulatory regions and enrichment analysis

**Fig. 4:**
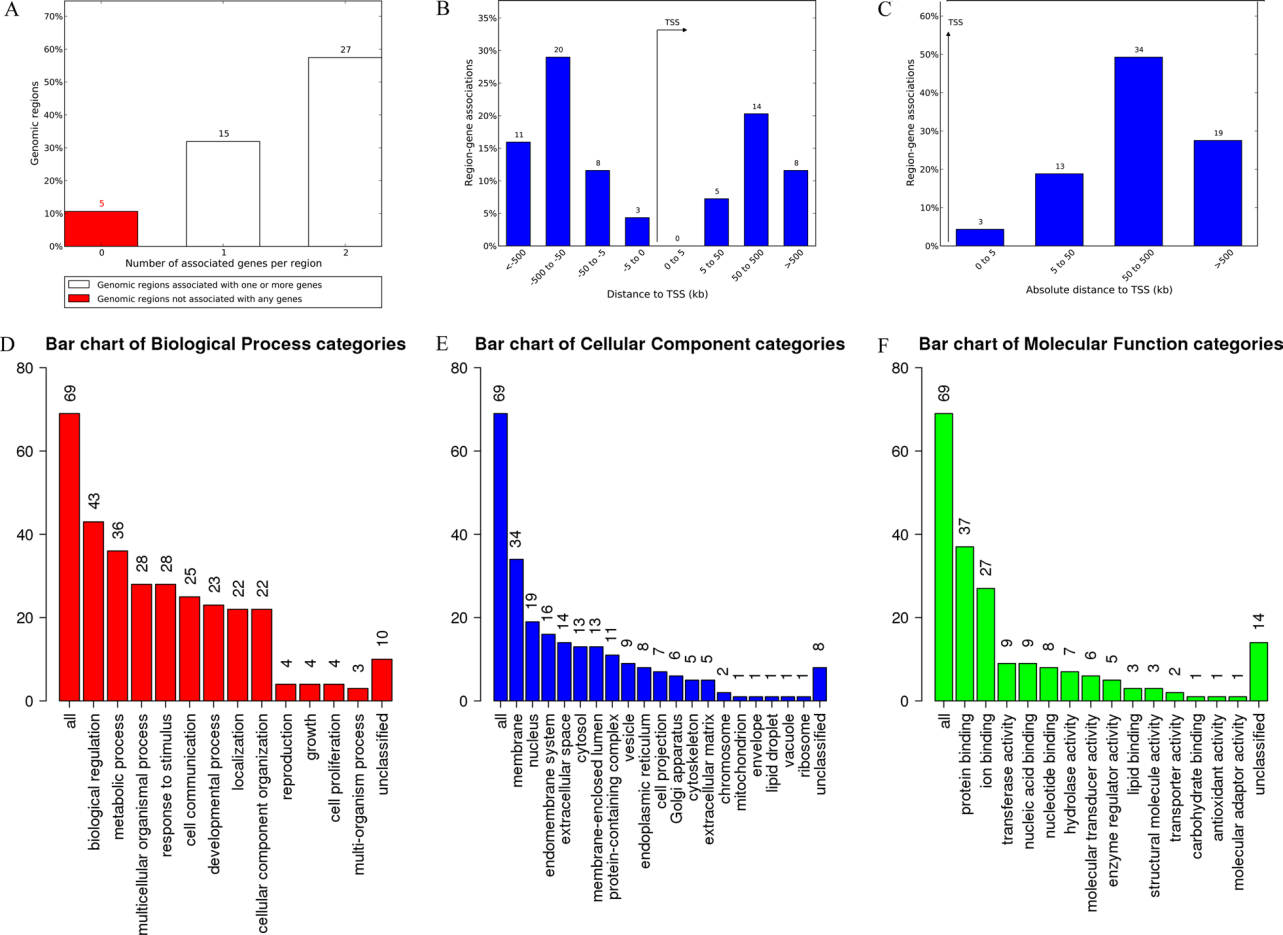
The genes associated with solo LTRs and GO Slim summaries. **A** The number of associated genes per solo LTR. **B** Binned by orientation and distance to TSS. **C** Binned by absolute distance to TSS. Biological process (**D**), cellular component (**E**), and molecular function (**F**) summaries are represented by red, blue, and green bars, respectively. The height of the bar represents the number of IDs in the gene list and in the category

GREAT analysis can predict possible regulated genes based on spatial proximity. The results describing the associations between each solo LTR and its putative regulated gene(s) are shown in Additional file 5: Table S3. A total of 69 genes were predicted. Among these, 5 solo LTRs were not associated with any genes, 15 solo LTRs were associated with 1 gene, and 27 solo LTRs were associated with 2 genes (Fig. 4A). The absolute distances of 3 genes to the transcription start site (TSS) were less than 5 kb. The absolute distances of 13 genes to the TSS were between 5 and 50 kb. The absolute distances of 34 genes to the TSS were between 50 and 500 kb. The absolute distances of 19 genes to the TSS were more than 500 kb (Fig. 4B, C).

To analyze the biological classification of key genes related to solo LTRs, GO Slim summaries were generated. The biological processes (BP) summary revealed that these genes were mainly enriched in biological regulation, metabolic process, multicellular organismal process, response to stimulus, cell communication, developmental process, localization, and cellular component organization (Fig. 4D). The GO Slim cellular component (CC) summary showed that these genes were significantly enriched in the membrane and nucleus, and the GO Slim molecular function (MF) summary showed that these genes were significantly enriched in protein binding and ion binding (Fig. 4E, F).

**Fig. 5:**
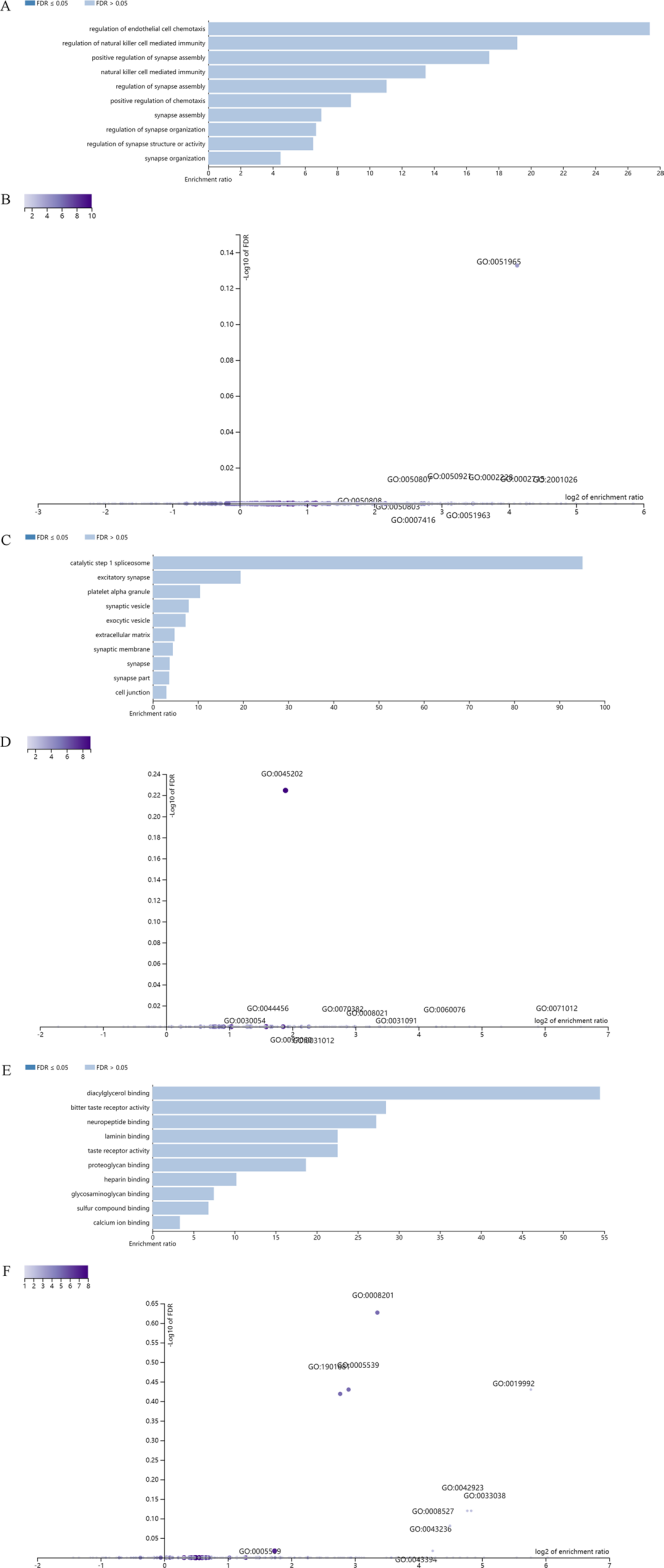
Enrichment result categories binned by biological process, cellular component, and molecular function. **A**, **B** Bar chart and customizable volcano plot of the biological process enrichment results. **C** and **D**, Bar chart and customizable volcano plot of the cellular component enrichment results. **E** and **F**, Bar chart and customizable volcano plot of molecular function enrichment results

Furthermore, these potential regulatory genes were all annotated to the selected functional categories and subjected to enrichment analysis. The top 10 most significant GO terms according to FDR value for BPs included the regulation of endothelial cell chemotaxis, the regulation of natural killer cell-mediated immunity, the positive regulation of synapse assembly, natural killer cell-mediated immunity, the regulation of synapse assembly, the positive regulation of chemotaxis, synapse assembly, the regulation of synapse organization, the regulation of synapse structure or activity, and synapse organization (Fig. 5A). The bar chart shows the enrichment ratio of the results. Bars representing categories with an FDR ≤ 0.05 are shown in a darker shade (Fig. 5A). The volcano plot in Fig. 5B shows the log2 of the FDR versus the enrichment ratio for all the functional categories in the database, highlighting the degree to which the significant categories are separated from the background. The size and color of a dot are proportional to the number of overlaps (for ORA). The significantly enriched categories are labeled, and the labels are positioned automatically by a force field-based algorithm at startup. The enrichment results for the CC and MF categories are illustrated in Fig. 5C–F. It must be noted that these results are entirely prediction-based and that future research is required to confirm any of the implied associations between solo LTRs and nearby genes.

Similar to the approach used for solo LTRs, GREAT prediction of genes putative regulated by proviral LTRs was also performed. Enrichment analysis was carried out as described for solo LTRs. The results describing the associations between each proviral LTR and its putative regulated gene(s) are shown in Additional file 6: Table S4. A total of 36 genes were predicted. Among these, 4 proviral LTRs were not associated with any genes, 6 proviral LTRs were associated with 1 gene, and 15 proviral LTRs were associated with 2 genes (Additional file 8: Figure S1A). Of these, the 4 proviral LTRs associated with no genes belong to two pairs of 5′ and 3′ LTRs of the same provirus, 2p12 and Yp11.2, respectively. In particular, 2p12 is a rather complete provirus. No genes with an absolute distance of less than 5 kb from the LTR to the TSS were found. The absolute distances to the TSSs were between 5 and 50 kb for 13 genes. The absolute distances to the TSSs were between 50 and 500 kb for 15 genes. The absolute distances to the TSSs were more than 500 kb for 8 genes (Additional file 8: Figure S1B, C). The GO Slim summaries for the biological classification of key genes related to proviral LTRs are shown in Additional file 8: Figure S1D–F. The enrichment results for BP, CC, and MF categories are shown in Additional file 9: Figure S2, Additional file 10: Figure S3, Additional file 11: Figure S4.

### In silico examination of the conserved transcription factor binding sites

**Fig. 6:**
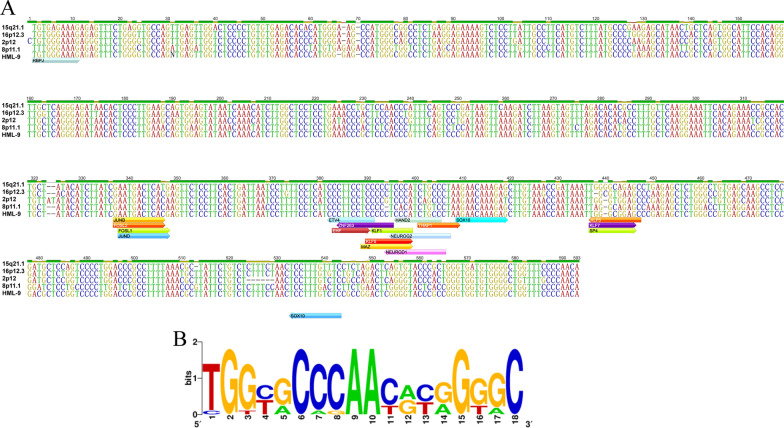
In silico examination of the conserved transcription factor binding sites and logos representing the PBSs of HML-9. **A** The forward arrow indicates the sense strand, and the reverse arrow indicates the antisense strand. Different transcription factors are marked with different colors. **B** PBS nucleotide sequence

Specific base insertions in HML-9 elements may influence the complexity of LTR transcriptional regulation [16]. A complete view of the putative transcription factor binding sites within the HML-9 LTR is shown in Fig. 6A. A total of 22 human transcription binding sites were predicted for 19 transcription factors: EHF, SOX10, FOS, FOSL1, FOSL2, JUNB, JUND, ETV4, KLF1, KLF5, KLF7, ZNF263, THAP1, SP4, RBPJ, HAND2, MAZ, NEUROG2, and NEUROD1. The motifs are marked on the sense strand and antisense strand of the consensus sequence.

### PBS type of HML-9 sequences

Traditionally, HERVs have been named according to the tRNA that binds their RT enzyme and PBS [59]. Thus, HERV-K is named after the lysine-tRNA. In the 11 proviral and consensus sequences of HML-9 elements analyzed, the PBS was located approximately 3–20 nucleotides downstream of the 5′LTR. To summarize the general variation of the PBS sequence within the HML-9 group, we generated a logo in which the letter height is proportional to the nucleotide conservation at each position (Fig. 6B). The results showed that the TGG starting nucleotides were the most conserved among the 18 bases. However, only the 15q21.1 and 8p11.1 PBSs belong to lysine, confirming the relatively low taxonomic value of this feature (Additional file 7: Table S5) [61, 62].

## Discussion

At present, the HML1-8 and HML10 groups have been characterized and identified [9, 16, 48, 49, 60–64]. However, existing research on HML-9 elements is very limited [44]. A descriptive study of HML-9 elements, especially the characterization and description of their features, is still lacking. Characterization of the genomic distribution of the HML-9 group is critical to understanding the regulation of the expression of this family in healthy humans and its relationship with diseases. Therefore, it is necessary to perform a systematic and comprehensive characterization of HML-9.

Our research followed the approach carried out in previously published studies [9, 48], completely mapping out the HML-9 proviruses and solo LTRs in the human genome and thus providing an exhaustive characterization of this group, including genomic distribution, structural characterization, phylogenesis, integration time analysis and regulatory function prediction. A total of 23 HERV-K HML-9 proviruses and 47 solo LTR elements were characterized. The chromosomal distribution of these proviruses and the solo LTRs revealed a nonrandom integration pattern. HERV-K HML-9 elements are usually enriched outside transcription units in the human genome [15, 65]. The results showed that these elements are mainly distributed in intergenic regions and introns. This may be because the integration of a HERV provirus within the transcription unit is harmful and therefore subject to negative selection and elimination during evolution [12, 15, 21–24]. In particular, the number of proviruses on the Y chromosome was significantly different from that predicted by the chi-square test (p = 0.01), which indicates that the male-specific region of the Y chromosome (MSY) accumulates higher densities of HERVs and associated sequences, consistent with previous studies [65].

Phylogenetic analyses showed that 5 sequences of HML-9 near-full-length proviruses as well as 10 *gag* elements, 8 *pro* elements, 11 *pol* elements, and 13 *env* sequences formed a unique monophyletic cluster, clearly divided from other HML groups, supported by the maximum bootstrap value. The phylogenetic trees of the *pro* and *pol* regions both revealed the presence of two well-supported clusters, identified here as HML-9 types a and b, which were statistically supported by bootstrap values of 100. The HML-9 type b cluster included the Dfam HML-9 reference, whereas the HML-9 type a cluster showed more divergence relative to the group references. In addition, the integration time of HML-9 proviruses was calculated using the LTR, *gag*, *pro*, *pol*, and *env* regions. The results indicated that the LTRs integrated between 17.5 and 48.5 mya. However, the main period of HML-9 integration based on 4 internal regions is between 37.5 and 151.5 mya. The difference in estimated integration time between the two methods likely occurred because internal coding regions can accumulate mutations during every replication cycle, while two identical LTRs integrate into the host genome during the integration phase [66]. Therefore, it is more reasonable to use LTRs to evaluate the integration time.

Furthermore, we performed prediction and cluster analysis of potential regulatory genes for both the HML-9 provirus and solo LTRs. A total of 69 genes were predicted. BP and MF analyses showed that these genes were associated with synapses. Previous studies have shown that HERV-W can interfere with neuronal protrusions and alter N-methyl-D-aspartate receptor (NMDAR)-mediated synaptic organization and plasticity through glia- and cytokine-dependent changes [67]. Here, our work suggested that HML-9 LTR-regulated genes may also be widely involved in the function of synapses. Furthermore, the prediction of TFBSs in HML-9 elements by JASPAR also indicated that HML-9 is likely to play a role in regulating downstream genes. In addition, for the PBS analysis of HML-9 elements, the results showed that the TGG starting nucleotides were the most conserved among the 18 bases. Similar to previous work [68, 69], we identified 11 proviral PBS sequences and confirmed that this nomenclature is imprecise because although HML-9 belongs to the HERV-K subgroup, only the PBSs of 15q21.1 and 8p11.1 belong to lysine. It should be noted that these results are entirely prediction based. Experimental validation studies are required to confirm the associations between these elements and these genes.

## Conclusion

A previous study of HML-9 (HERVK14C) indicated that HML-9 could exert its effects in different tissues under physiological conditions as well as during disease development, possibly contributing to immune regulation and antiviral defense [44]. To systematically study the important role of HML-9 in pathological and physiological processes, the current work provides a clear and detailed description of all HML-9 elements integrated into the human genome, which could contribute to better defining the real impact of these elements and their contribution to the genome.

## Supplementary Information

**Additional** **file** **1:** **Dataset** **S1.** The nucleotide sequence of HML-9 proviral elements.

**Additional** **file** **2:** **Dataset** **S2.** The nucleotide sequence of HML-9 solo LTR elements.

**Additional** **file** **3:** **Table** **S1.** HML-9 proviral sequences colocalized with genes.

**Additional** **file** **4:** **Table** **S2.** HML-9 solo LTR sequences colocalized with genes.

**Additional** **file** **5:** **Table** **S3.** The associations between each solo LTR and its putative regulated gene(s).

**Additional** **file** **6:** **Table** **S4.** The associations between each proviral LTR and its putative regulated gene(s).

**Additional** **file** **7:** **Table** **S5.** PBS types in HML-9.

**Additional** **file** **8:** **Figure** **S1.** The genes associated with proviral LTRs and GO Slim summaries. **A** The number of associated genes per proviral LTR. **B** Binned by orientation and distance to TSS. **C** Binned by absolute distance to TSS. Biological process (**D**), cellular component (**E**), and molecular function (**F**) summaries are represented by red, blue, and green bars, respectively. The height of the bar represents the number of IDs in the gene list and in the category.

**Additional** **file** **9:** **Figure** **S2.** The enrichment results for the biological process. **A** The bar chart plots the enrichment results vertically with the bar width equal to the enrichment ratio in ORA. **B** Customizable volcano plot. The inset shows an initial layout for comparison.

**Additional** **file** **10:** **Figure** **S3.** The enrichment results for cellular component. **A** The bar chart plots the enrichment results vertically with the bar width equal to the enrichment ratio in ORA. **B** Customizable volcano plot. The inset shows an initial layout for comparison.

**Additional** **file** **11:** **Figure** **S4.** The enrichment results for molecular function. **A** The bar chart plots the enrichment results vertically with the bar width equal to the enrichment ratio in ORA. **B** Customizable volcano plot. The inset shows an initial layout for comparison.

## Data Availability

All data generated or analyzed during this study are included in this published article.

## Abbreviations

HERVs: Human endogenous retroviruses
TEs: Transposable elements
REs: Retroelements
LTRs: Long terminal repeats
PBS: Primer binding site
HML: Human MMTV-like
MSY: Male-specific regions of the Y chromosome
RT: Reverse transcriptase
ML: Maximum likelihood
NNI: Nearest neighbor interchange
GREAT: Genomic regions enrichment of annotations tool
WebGestalt: WEB-based Gene SeT AnaLysis Toolkit
ORA: Over-representation analysis
GSEA: Gene set enrichment analysis
ORFs: Open reading frames
Mya: Million years ago
TSS: Transcription start site
BP: Biological processes
CC: Cellular component
MF: Molecular function
FDR: False discovery rate
