# Supplementary figures and images for: Comprehensive identification and characterization of the HERV-K (HML-9) group in the human genome
Source: Retrovirology. 2022 Jun 8;19:11. doi: 10.1186/s12977-022-00596-2 (PMC9178832; doi:10.1186/s12977-022-00596-2)

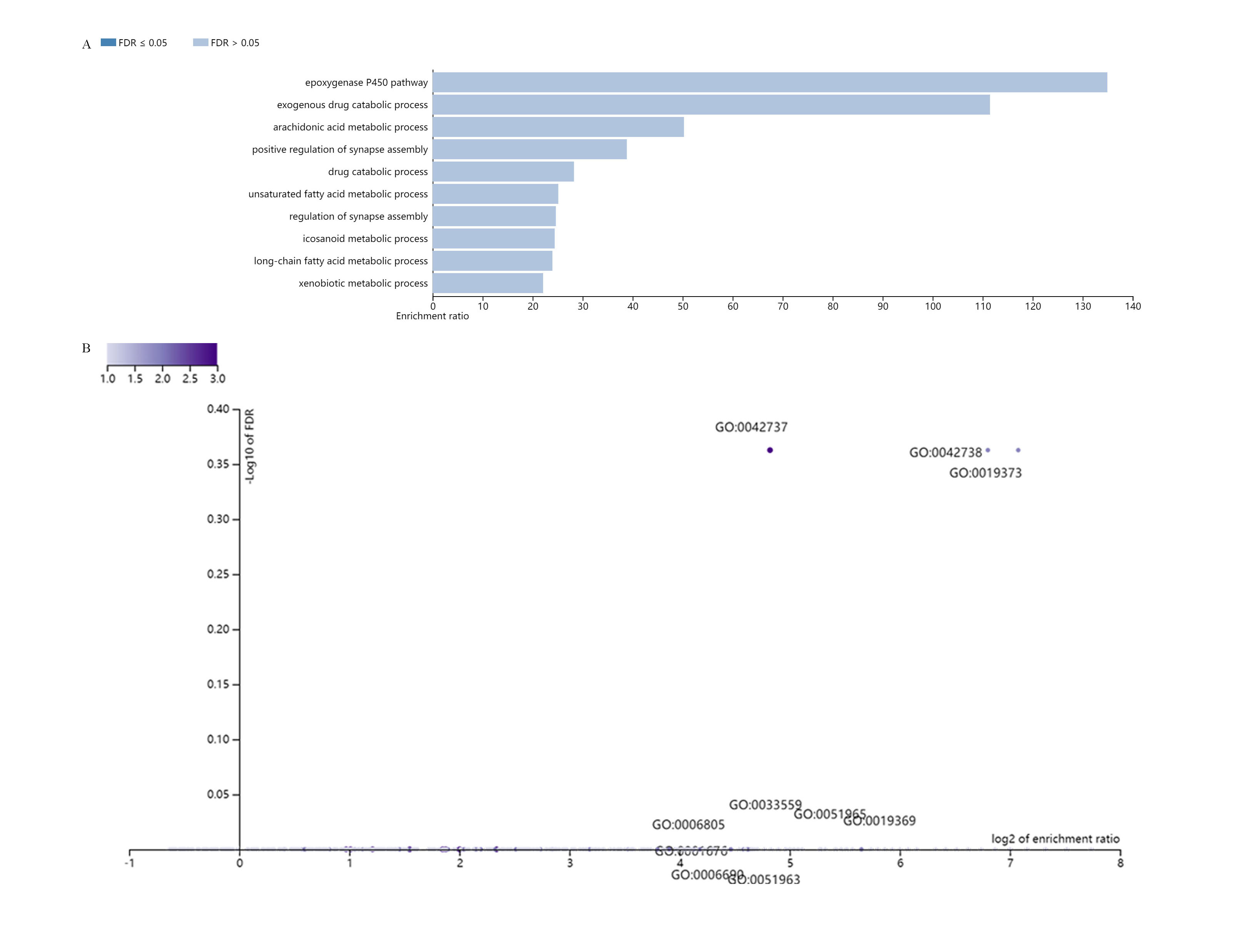

Supplement: Supplementary file 9 — Additional file 9: Figure S2. The enrichment results for the biological process. A The bar chart plots the enrichment results vertically with the bar width equal to the enrichment ratio in ORA. B Customizable volcano plot. The inset shows an initial layout for comparison. [file 12977_2022_596_MOESM9_ESM.tif]

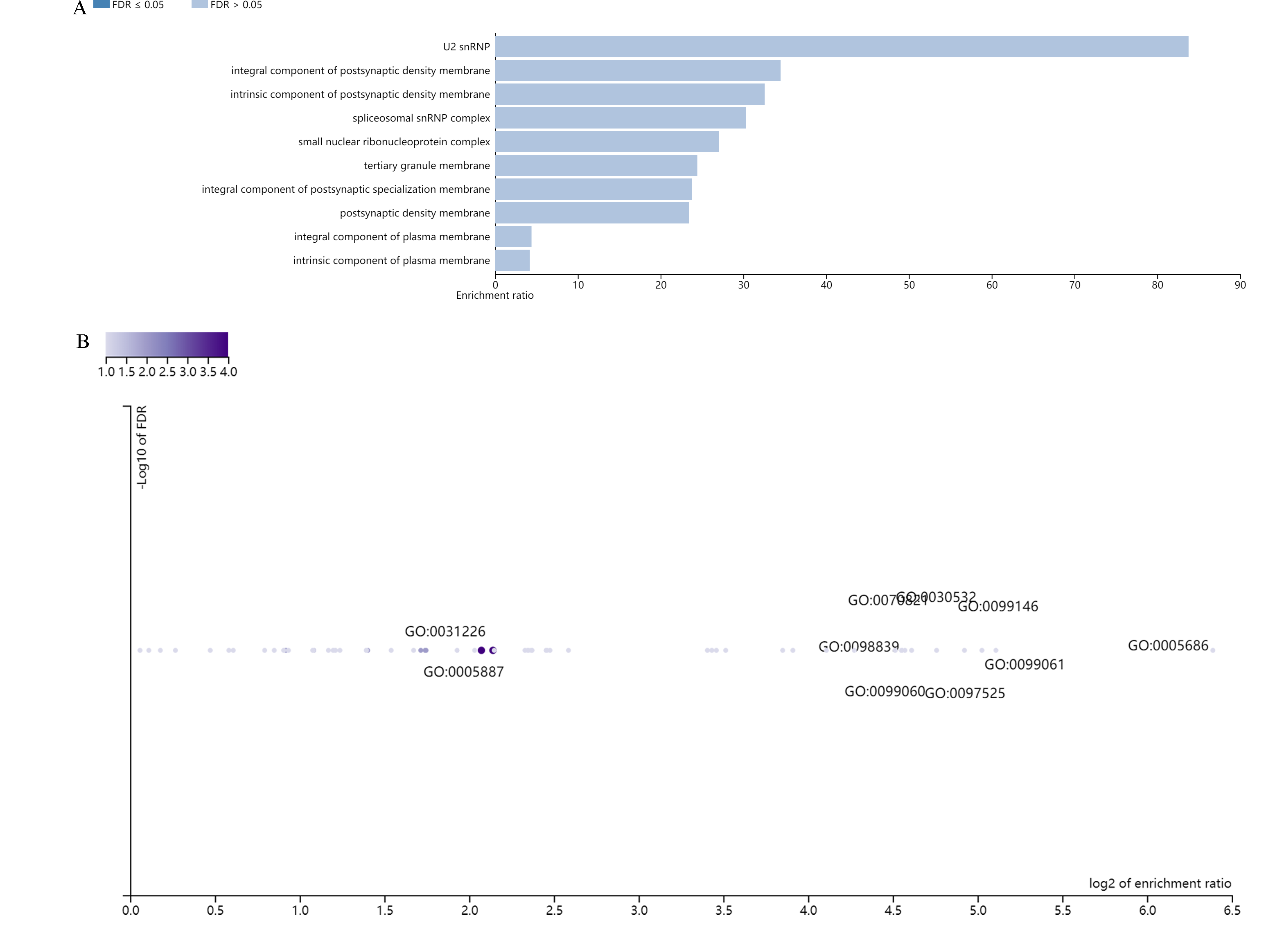

Supplement: Supplementary file 10 — Additional file 10: Figure S3. The enrichment results for cellular component. A The bar chart plots the enrichment results vertically with the bar width equal to the enrichment ratio in ORA. B Customizable volcano plot. The inset shows an initial layout for comparison. [file 12977_2022_596_MOESM10_ESM.tif]

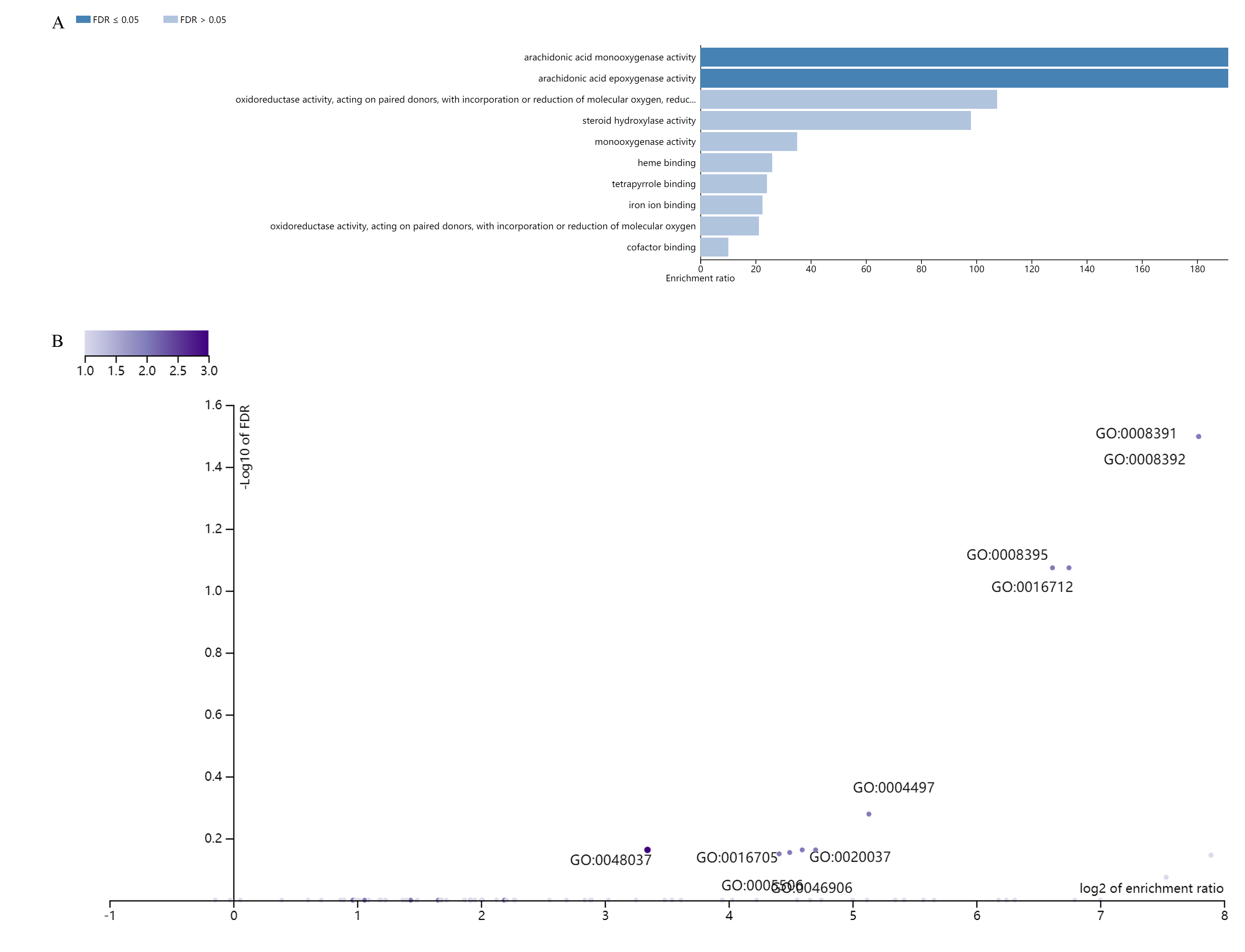

Supplement: Supplementary file 11 — Additional file 11: Figure S4. The enrichment results for molecular function. A The bar chart plots the enrichment results vertically with the bar width equal to the enrichment ratio in ORA. B Customizable volcano plot. The inset shows an initial layout for comparison. [file 12977_2022_596_MOESM11_ESM.tif]
